# Stability of executive function and predictions to adaptive behavior from middle childhood to pre-adolescence

**DOI:** 10.3389/fpsyg.2014.00331

**Published:** 2014-04-22

**Authors:** Madeline B. Harms, Vivian Zayas, Andrew N. Meltzoff, Stephanie M. Carlson

**Affiliations:** ^1^Institute of Child Development, University of MinnesotaMinneapolis, MN, USA; ^2^Department of Psychology, Cornell UniversityIthaca, NY, USA; ^3^Institute for Learning and Brain Sciences, University of WashingtonSeattle, WA, USA

**Keywords:** executive function, affective decision-making, event-related potentials, adolescence, reward processing

## Abstract

The shift from childhood to adolescence is characterized by rapid remodeling of the brain and increased risk-taking behaviors. Current theories hypothesize that developmental enhancements in sensitivity to affective environmental cues in adolescence may undermine executive function (EF) and increase the likelihood of problematic behaviors. In the current study, we examined the extent to which EF in childhood predicts EF in early adolescence. We also tested whether individual differences in neural responses to affective cues (rewards/punishments) in *childhood* serve as a biological marker for EF, sensation-seeking, academic performance, and social skills in early adolescence. At age 8, 84 children completed a gambling task while event-related potentials (ERPs) were recorded. We examined the extent to which selections resulting in rewards or losses in this task elicited (i) the P300, a post-stimulus waveform reflecting the allocation of attentional resources toward a stimulus, and (ii) the SPN, a pre-stimulus anticipatory waveform reflecting a neural representation of a “hunch” about an outcome that originates in insula and ventromedial PFC. Children also completed a Dimensional Change Card-Sort (DCCS) and Flanker task to measure EF. At age 12, 78 children repeated the DCCS and Flanker and completed a battery of questionnaires. Flanker and DCCS accuracy at age 8 predicted Flanker and DCCS performance at age 12, respectively. Individual differences in the magnitude of P300 (to losses vs. rewards) and SPN (preceding outcomes with a high probability of punishment) at age 8 predicted self-reported sensation seeking (lower) and teacher-rated academic performance (higher) at age 12. We suggest there is stability in EF from age 8 to 12, and that childhood neural sensitivity to reward and punishment predicts individual differences in sensation seeking and adaptive behaviors in children entering adolescence.

## Introduction

Executive function (EF) is comprised of a constellation of functions involving the control of thought and action, including the abilities to inhibit pre-potent responses, flexibly shift attention, and update information in working memory (Miyake et al., 2000; Miyake and Friedman, 2012). Recent literature has distinguished between cool EF, which involves the execution of these processes under relatively neutral conditions, and hot EF, which occurs in emotionally salient contexts that may also require risk and reward processing. Cool and hot aspects of EF show protracted maturation across development and may contribute to real-world behavior in different and/or overlapping ways (Zelazo and Carlson, 2012). The goal of the present work was to examine the influences of hot and cool EF and their neural correlates in childhood on adaptive behavior around the transition to adolescence.

EF is readily measurable during the preschool period, especially between ages 3 and 5, but improved performance in EF tasks is seen well into adolescence (for overview see Carlson et al., 2013). The gradual maturation of EF is likely due to the necessity of prefrontal cortex (PFC) engagement, particularly of the dorsolateral region, to perform these high-level cognitive processes (Bunge and Zelazo, 2006). Children as young as 6 years have been shown to activate the PFC when completing EF tasks, but they show a more diffuse network of activation than adults, which suggests that this network gains efficiency with development (Casey et al., 2000). Structurally, the PFC matures slowly across development; indeed, synaptic pruning of this region does not begin in earnest until adolescence (Casey et al., 2000). Behavioral research suggests that EF skills do not reach their full capacity until early adulthood (Steinberg et al., 2008; Zelazo et al., 2013).

As mentioned, two differentiable but related categories of EF—“cool EF” and “hot EF”—have been proposed based on the level of contextual emotional salience. Experimental tasks have been developed to assess both hot and cool EF. Classic cool EF tasks often involve performing mental operations on neutral stimuli. For example, in the flanker task (Eriksen and Eriksen, 1974; Rueda et al., 2004), individuals identify the direction of a target stimulus (an arrow) that is “flanked” by distracters facing the opposite direction. Likewise, in the Dimensional Change Card Sort task (DCCS; Zelazo, 2006), individuals sort bivalent stimuli on one dimension and then switch to the other (e.g., sort by color then by shape).

In contrast, Hot EF tasks involve performing mental operations in motivationally salient contexts or on motivationally salient stimuli. For example, in Mischel et al.'s (1989) classic delay of gratification task, children must refrain from eating a tempting treat or ringing a bell that would end the delay period in order to receive a larger reward. Likewise, in affective decision-making or gambling tasks, individuals make decisions about risks and potential rewards. In the Iowa Gambling Task, for example, participants choose among four options on each trial, each of which yields either long-term advantages or disadvantages and either short-term rewards or punishments (Bechara, 2004). This task thus involves learning about the most advantageous option in the context of risks and rewards. Although classified in the same “hot EF” category as delay tasks, gambling tasks recruit different cognitive processes and do not always correlate with delay tasks in children (Hongwanishkul et al., 2005). However, because children beyond the preschool period have little difficulty waiting for a reward, gambling tasks are the most paradigmatic method for examining hot EF in older children and adolescents.

There is some controversy as about the degree to which cool and hot EF tasks rely on overlapping vs. dissociated cognitive and neural processes. Using behavioral evidence, some researchers find associations in performance on cool and hot EF tasks (e.g., Carlson and Moses, 2001) whereas others find dissociations (Hongwanishkul et al., 2005; Smith et al., 2012). In a large sample of preschoolers, Carlson et al. (2014) found support for separate but related Conflict (cool) and Delay (hot) factors in a confirmatory factor analysis. At a neural level, by one account, hot and cool EF tasks rely on the same basic circuitry in PFC, but hot EF tasks are more difficult due to the bottom-up affective factors (primarily from reward-sensitive ventral striatum) that must be overcome (Prencipe et al., 2011, see also Reyna and Zayas, 2014). Another account, based on lesion data, suggests more fundamental differences between hot and cool EF, with the former relying primarily on orbitofrontal cortex (Bechara, 2004) and the latter relying on dorsolateral PFC (Casey et al., 2000).

The tasks that are chosen likely play a role in conflicting findings. For example, affective decision-making in a gambling task requires more updating than a delay of gratification task, and thus gambling tasks may relate more strongly to measures of cool EF (Hongwanishkul et al., 2005). In addition, performance on hot gambling tasks tends to lag significantly behind performance on cool EF tasks (Hooper et al., 2004; Prencipe et al., 2011). This may be due to more delayed maturation of the neural circuitry involved in emotion regulation and/or greater sensitivity to affective cues in younger children.

Regardless of the neural mechanisms involved, individual differences in hot and cool EF tend to be persistent over time. This is especially true in preschoolers, who show a high level of stability in their relative performance on both conflict and delay of gratification EF tasks (Carlson et al., 2004; Hughes and Ensor, 2007). We know less about the stability of individual differences in EF beyond the preschool period, but the extant research suggests that individual differences in EF tend to persist over time. For example, two studies (Eigsti et al., 2006; Casey et al., 2011) have found that the proportion of time preschoolers directed their attention away from rewarding stimuli during a delay-of-gratification task predicted their reaction times in a go-no-go task many years later.

### Neural correlates of hot EF development

Due to the limitations of scanning young children, the majority of our knowledge about the neural bases for the development of EF comes from studies examining electrical event-related potentials (ERPs) recorded during EF tasks. One component of interest, the P300, is a stimulus-locked component thought to be generated from frontal and temporal-parietal regions, and to be involved in updating working memory and inhibition (Polich, 2007). The P300 is seen approximately 300 ms post-stimulus in adults, but is delayed to 800–1200 ms post-stimulus in children (Tucker, 1993), suggesting increasing efficiency of EF networks with age.

A classic P300 paradigm is an oddball task, in which participants respond to a rare target among many distracters, but it is well established that the P300 is elicited by EF tasks as well. For example, in a flanker task, the P300 has higher amplitude after incongruent vs. congruent trials, suggesting this component might reflect inhibition of extraneous stimulus processing (Tucker, 1993). The P300 has also been found in the context of a hot EF task in 8-year-old children (Carlson et al., 2009). On a child version of the Iowa Gambling Task, the P300 had a higher amplitude after punishment than after reward trials, and the amplitude difference between loss and reward trials predicted children's performance on the task: Those who showed a more pronounced P300 response to losses vs. rewards learned to avoid disadvantageous and high-frequency punishment choices to a greater extent over the course of the task. In this case, the P300 served as a neural signature of focusing attention on a stimulus that provides important information about whether something should be approached or avoided. In the context of this task, greater sensitivity to punishment led to more avoidance of bad plays, and thus, better performance.

Another component of interest is the stimulus-preceding negativity, or SPN. This component occurs after a response has been made and just before feedback occurs. The SPN has recently been measured in children and seems to occur in the context of reward-based tasks. For example, Stavropoulos and Carver (2013) reported an SPN in 6- to 8-year-old children during a reward-based guessing game. They found larger SPN amplitudes for rewards that were accompanied by a smiling face than those accompanied by a scrambled face, suggesting that social stimuli were perceived as more salient. Although the Stavropoulos and Carver (2013) study involved no punishment, when negative feedback does occur, the SPN tends to be larger prior to receiving negative than positive feedback. This pattern has been found in school-age children for both a probabilistic learning task (Groen et al., 2007) and a gambling task (Carlson et al., 2009). Because research indicates that people are generally more sensitive to punishment than reward (Vaish et al., 2008), these results suggest that the SPN may reflect the emotional salience of an anticipated stimulus. Indeed, the SPN appears to be generated by the insular cortex and may reflect dopaminergic activity there (Bocker et al., 1994). As with the P300, the SPN may also be a neural signature of learning from feedback (Carlson et al., 2009). Given the probable link between SPN and risk and reward-processing, this component could be a particularly informative neural signature to examine prior to adolescence. Children who are more sensitive to anticipated punishment than anticipated reward as reflected by the SPN might show better adaptive outcomes in adolescence.

### Individual differences in EF and adaptive behavior in adolescence

Adolescence is a time of significant cortical reorganization, potentially even a sensitive period during which current developmental trajectories can be reinforced or re-directed. Many of the neurobehavioral changes that take place during adolescence may be influenced by changes in hormone levels associated with puberty (Steinberg, 2005). At the same time, there is a dramatic change in the context in which teenagers function. Moving from elementary school to middle and high school involves adapting to new peer groups, increased academic expectations, and increased exposure to high-risk activities. Given the high level of flux in the brain, body, and environment, it is unsurprising that behavior problems often emerge for the first time in adolescence.

Adolescents are more likely than older and younger individuals to engage in risky behavior such as the use of illegal drugs (Substance Abuse and Mental Health Services Administration, 2007) and engagement in unsafe sex (Finer and Henshaw, 2006). However, adolescents do not appear to evaluate the risks or consequences of behavior differently than adults in hypothetical situations (e.g., Beyth-Marom et al., 1993), suggesting that the PFC functions adequately in “cool” contexts that are not emotionally salient. Rather, behavioral differences in adolescence are more marked by the “heat of the moment” when a risky decision is made.

Developmental models characterize human adolescence as a period of increased risk-taking due to immature EF/PFC development, which is not yet up to the challenge of coping with more active reward-processing circuitry (i.e., ventral striatum) (Galvan et al., 2006; Ernst et al., 2009; Steinberg, 2010). It is noteworthy that substantial reorganization of the neural systems underlying EF takes place during adolescence. In the PFC, gray matter reaches peak thickness in early adolescence, and is pruned during the next few years (Paus, 2005). In addition, connectivity between limbic and pre-frontal brain regions increases substantially during adolescence (Eluvathingal et al., 2007). The implications of these changes are that by the end of adolescence, the PFC operates more efficiently and there is greater coordination between pre-frontal and limbic systems.

Behavioral and neuroimaging research suggests that increased risk-taking by adolescents may stem from a greater sensitivity to potential rewards than in other age groups. For example, in affective decision-making tasks such as the Iowa Gambling Task, adolescents are more approach-oriented than are pre-adolescents or adults. One study found that although both adults and adolescents played increasingly more from advantageous decks over the course of the task, only adults decreased their plays from disadvantageous decks (Cauffman et al., 2010). Furthermore, in an fMRI study, Galvan et al. (2006) found that adolescents activated the reward-sensitive nucleus accumbens more than children or young adults during a reward-processing task, and activated the OFC, which is thought to play a regulatory role in risk and reward processing, less than adults. Thus, appetitive reward-sensitive systems may mature earlier in adolescence than regulatory systems, possibly contributing to the observed increase in risky behavior (Ernst et al., 2009).

Despite the reported increase in risky behavior among adolescents, substantial individual differences exist. Better EF skills could be a protective factor that reduces risk-taking behavior in adolescents. Research shows that childhood EF, particularly hot EF, predicts a variety of outcomes in adolescence. Seminal work by Mischel et al. (1989; reviewed in Zayas et al., 2014) re-evaluated high-school students who had completed the delay of gratification task during preschool, and found that individuals who refrained from eating a desirable treat and waited 15 min for a larger reward scored significantly higher on their SATs than those who did not wait, independent of IQ assessed at age 4. In addition, parents rated the adolescents who had delayed as preschoolers higher in social cognitive skills and emotional coping. In further follow-up studies, delay of gratification at age 4 predicted more efficient EF (Eigsti et al., 2006) and less interference on a social-reward version of a go-nogo task at the behavior and neural (fMRI) levels (Casey et al., 2011). Other research has found longitudinal relations between preschool delay of gratification performance and physical health. Children who had settled for a lesser reward at age 4 were 30% more likely to be overweight at age 11 (Seeyave et al., 2009). Thus, the ability to delay gratification in childhood appears to reflect individual differences that influence the development of many aspects of adaptive behavior.

In contrast to the delay of gratification task, there is less longitudinal evidence linking performance on hot affective decision making tasks with later EF and life outcomes. Nonetheless, extant work suggests that affective decision-making tasks could prove useful in evaluating propensities toward risk-taking, particularly in adolescence. Adolescents both engage in more sensation seeking behavior than younger children and adults and make more decisions based on reward rather than punishment feedback (Cauffman et al., 2010; Albert and Steinberg, 2011). These patterns might derive from the same mechanisms, such as dopaminergic activity in the brain (e.g., Ernst et al., 2009). Such mechanisms presumably operate earlier in development as well, in which case it may be possible to assess the risk for future sensation seeking behavior by examining behavioral and neural sensitivity to reward and punishment at earlier ages.

Unlike delay tasks, gambling tasks tap into risk as well as reward processing and may involve larger cognitive demands (Hongwanishkul et al., 2005). Research suggests that the ability to optimize one's gambling strategy develops sometime after the emergence of initial cool EF skills (Hooper et al., 2004; Prencipe et al., 2011). Based on simplified versions of the Iowa Gambling Task, young children seem unable to optimize long-term outcomes, responding only to immediate losses or gains. Not until sometime between middle childhood and early adolescence do children begin to integrate the frequency of gains and losses with long-term consequences (Huizenga et al., 2007; Carlson et al., 2009). Research also suggests that from childhood to adolescence, individuals become more physiologically sensitive to the *anticipation* of gains and losses, showing larger skin conductance responses *before* choosing frequent loss doors at age 16–18 than at age 10–14 (Crone and van der Molen, 2007). Although children may perform as well as adults at these ages, they still show some differential brain activity; for example, 9–12 year-old children have been shown to activate the anterior cingulate cortex (ACC), which is involved in error monitoring, more than adults on high-risk trials. This finding suggests that the task may be more effortful for them (Van Leijenhorst et al., 2006).

In addition to performance indices, individual differences in neural responses during affective decision-making tasks have also been found to predict later behavioral outcomes. In an ERP study of adolescent monozygotic twins, the P300 effect (amplitude of the P300 in loss vs. gain trials) predicted later alcohol abuse: In each twin pair, one individual began to abuse alcohol in adulthood, and these individuals tended to have had lower amplitude P300 responses to loss trials in early adolescence (Carlson et al., 1999). This study suggests that a blunted P300 effect could be an endophenotype for later high-risk behavior. In the current study, one goal was to extend this finding to examine the extent to which neural responses during affective decision making predict less extreme forms of sensation seeking in a low-risk sample. A related goal, given the literature linking preschool EF to life success, was to assess the extent to which both hot and cool EF predict other adaptive outcomes, such as academic performance and social skills.

### Present study

The overarching goal of our research was to characterize individual differences in hot and cool EF that might lead individuals to divergent pathways in adolescence. We re-contacted a cohort of typically-developing children who had been assessed at age 8 on both cool EF measures and a relatively hot EF measure (gambling task) when they were 12 years old and entering adolescence. This longitudinal study had two specific aims: (i) to assess the stability of individual differences in cool EF from middle childhood to early adolescence, an age period that has not yet been the focus of longitudinal research on EF, and (ii) to examine the degree to which cool EF, as well as affective decision-making and its neural correlates at age 8 years (middle childhood), predicted adaptive behavior (academic performance, social skills, and sensation seeking) at age 12 years. We chose Flanker and DCCS tasks to examine cool EF, and a child-friendly gambling task to examine hot EF/affective decision-making, as these are the most paradigmatic and well-supported tasks in the literature to measure these constructs. We hypothesized there would be long-term stability of individual differences in EF. With respect to adaptive behavior, we hypothesized that better performance on an affective decision-making task and/or neural correlates of sensitivity to reward and punishment would predict higher academic achievement and social adjustment and lower sensation-seeking in pre-adolescence. This is the first study, to our knowledge, to examine longitudinal correlates of both cool EF and a hot affective decision-making task in this age group.

## Materials and methods

### Time 1

#### Participants

Eighty-four children who were recruited by telephone from the University of Washington (Seattle) participant database when they were 8 years old completed a series of EF tasks. Here, we report data from 78 children (37 males, 41 females) who participated at both age 8 and age 12. This sample had a mean age of 8 years, 4 months (*SD* = 8 months) at Time 1. Participants were primarily white/non-Hispanic. Maternal education (mode) was a 4-year college degree. Written consent from parents and verbal assent from children was obtained.

#### Procedure

Participants were tested in a laboratory by a female experimenter. All tasks, other than the Peabody Picture Word Vocabulary Test (PPVT-4), were administered on a computer using E-prime software. An electrode sensor cap (Neuroscan 21-channel) was placed on the child's head while they were seated in front of a computer monitor. A chin rest controlled the distance and alignment to the monitor. During the tasks, participants responded by clicking response-specific buttons on a keyboard. Children completed the following four tasks.

***Attention network task (Rueda et al., 2004)***. On this flanker-type task, participants were shown a row of fish and asked to quickly and accurately indicate whether the *central* fish points to the right or left by a key press. The surrounding “flanker” fish pointed in either the congruent or incongruent direction compared to the central fish (50% of trials each). A spatial cue appeared 150 ms before the preceding the target stimulus (central fish) and was presented in the center, top, or bottom of the screen (48 trials each). The target stimulus always appeared in the center of the screen, 450 ms after the offset of this cue. ITIs varied from 400 to 1600 ms. Participants completed 1 practice block of 24 trials and 4 blocks of 48 trials for data collection. Feedback after each trial was given only in the practice condition. Mean accuracy and median reaction times for congruent and incongruent trials were scored.

***Dimensional change card sort (adapted from Zelazo, 2006)***. This task required participants to shift between sorting stimuli by shape or by color. Participants completed one 40-trial block of practice trials in which only the dominant cue (shape) was presented and four 40-trial blocks of test trials which included 75% dominant (shape) trials and 25% non-dominant (color) trials. Each trial consisted of two target stimuli presented in the upper left (red star) and upper right (blue square) corner of the screen. At the start of the trial, a cue “SHAPE” or “COLOR” appeared in the middle of the screen for 1000 ms, along with a test stimulus directly below it. Participants' task was to match the test stimuli (a red square or blue star) to one of the two target stimuli on the dimension (shape or color) indicated by the cue using a key press. The ITI was 1000 ms during which a gray fixation cross appeared in the middle of the screen. No error feedback was given on any of the test trials. Instructions were presented on the computer screen and described to each participant by a female experimenter. Mean accuracy and median reaction times were scored.

***Hungry donkey gambling task (HDT) (Crone and van der Molen, 2004)***. The objective of this task was to win as many apples for the donkey as possible. Before the game, children were shown a prize bin and told that they could select a prize if they gained more apples than they lost (but all were invited to select a prize at the end). In the task, there were four doors participants could choose to open by pressing the corresponding key, among which long-term gains were crossed with frequency of loss. Door A was disadvantageous over time and yielded frequent small losses (8–12 apples lost on 50% of trials), door B was disadvantageous and yielded infrequent large losses (50 apples on 10% of trials), door C was advantageous but yielded frequent miniscule losses (1–3 apples on 50% of trials), and door D was advantageous and yielded infrequent small losses (10 apples on 10% of trials). Doors A and B yielded a net loss of 10 apples and doors C and D yielded a net gain of 10 apples over the course of the task. Gain and loss information was presented on each trial 500 ms after door selection as a column of red apples crossed out (losses) and a column of green apples (gains). This information remained on the screen for 1000 ms. Overall gains and total number of losses were scored across 280 trials, which were split into 4 blocks of 70 trials. The total number of trials in which a net loss (more apples lost than gained) was incurred was used as a performance measure. For more details on this task, see Carlson et al. (2009).

***PPVT-4 (Dunn and Dunn, 2007)***. Children completed this task for an approximation of their verbal IQ. On each trial, the experimenter said a word and children were asked to indicate the corresponding picture from four options. Age-standardized scores were obtained.

#### EEG recording

Continuous EEG was recorded from 21 channels during the HDT using a Neuroscan net. Electrodes were placed over the left and right prefrontal (Fp1, Fp2), frontal (F3, F4), inferior frontal (F7, F8), temporal (T7, T8), central (C3, C4), parietal (P3, P4), posterior parietal (P7, P8), occipital (O1, O2), and three midline locations (Fz, Cz, Pz). An electrode placed over the left mastoid was used as the online reference for other channels. A NuAmp 40 Channel Neuroscan amplifier was used with a sampling frequency of 1000 Hz and an online band-pass filter of 0.10–200 Hz. EEG activity was filtered offline using a 30 Hz low-pass filter and re-referenced using an average reference of the right and left mastoid electrodes. Trials contaminated by excessive eye movement or muscle artifacts (150 mV from baseline) were excluded. ERP data from 78 children were included in the analysis. For further details, see Carlson et al. (2009).

#### ERP analysis

We focused on two ERP components from the HDT, the post-outcome P300 and pre-outcome SPN. We calculated the P300 effect for each participant by subtracting the average amplitude (area under the curve) of trials in which a net loss was incurred from the average amplitude of trials in which a net reward was incurred during the period 300–800 ms post-feedback. We calculated the pre-outcome anticipation of loss effect by subtracting mean voltage for the SPN (−150 ms preceding feedback to +50 ms post-feedback) for high-frequency-punishment door selections (doors A and C) from mean voltage for low-frequency-punishment door selections (doors B and D). Positive numbers, therefore, indicate larger (more negative-going) anticipation effects (see Carlson et al., 2009). A minimum of 20 artifact-free trials for each trial type involved in the calculation was used to calculate P300 and SPN effects. Using difference scores for both these components ensures that signal-to-noise ratios are equated across participants despite individual differences in children's distribution of door choices.

### Time 2

#### Participants

Families who participated at Time 1 (8 years old) were mailed an invitation to participate in a follow-up study 4 years later, along with questionnaire packets and instructions for completing games online. Of these, 78 families (37 males, 41 females) sent back child and parent questionnaires. The mean age of our Time-2 sample was 12 years, 4 months (*SD* = 9 months) and in 6th or 7th grade (both grades are in middle school in the Seattle area). 66 children (31 males, 35 females) sent back teacher-completed questionnaires, and 67 (32 males, 35 females) completed the online games.

#### Procedure

Child participants and their parents were mailed separate packets of questionnaires (with separate self-addressed return envelopes) so that children could keep their responses private from their parents (and this was suggested in instructions to both children and parents). Written consent was obtained from parents and written assent from children. Parents were asked to give the teacher version of the Social Skills Improvement System to their child's teacher in the humanities and/or math. Packets also included instructions on how to access the online EF tasks. Children were instructed to complete the tasks when they were alone and free from distractions.

#### Questionnaires

Participants and their parents and teachers completed a battery of questionnaires assessing social skills, sensation seeking, and academic performance.

***Social skills and academics***. *The Social Skills Improvement System* (SSIS; Gresham and Elliot, 2008) assessed children's social functioning in everyday life and was completed by parents, children, and teachers separately (three versions created by the developers). The form contains 75–85 questions, depending on the informant. Questions (e.g., “Takes responsibility for part of a group activity.”) are rated on a 4-point scale (never, sometimes, often, almost always). Subscales for social skills (46 items), problem behaviors (30 items), and academic competence (teacher form only) are included. For the academic competence scale, teachers rated how children ranked among their peers on a 5-point scale (ranging from lowest 10% to highest 10%) for 7 items that queried specific academic skills, motivation, and intellectual ability. Internal consistency alphas for each subscale of this form are 0.93–0.96 (Gresham and Elliot, 2008). For the current study, we included child and teacher reports in our analyses. Higher scores on the social skills and academic performance scales indicate better performance. We do not report on problem behaviors, because teachers reported very low levels of problem behaviors in this sample (mean 8.7, whereas rating “sometimes” for each item would score 30).

***Sensation seeking***. Children completed the *Sensation Seeking Scale for Children* (SSSC; Russo et al., 1993). In this 26-item form, children are asked to choose between two alternatives, e.g., “I don't do anything I might get in trouble for” vs. “I like to do new and exciting things, even if I think I might get in trouble for doing them.” The form has subscales for thrill and adventure seeking, drug and alcohol seeking, and social disinhibition. We collected data on the full questionnaire, but used only the thrill and adventure subscale (12 items) for analyses because children in this sample reported little sensation seeking in the other two categories. The internal consistency alpha reported for this subscale is 0.81 (Russo et al., 1993). Scores for this subscale were summed, and higher scores reflect higher levels of sensation seeking.

***Online EF games***. Participants completed online, computerized versions of the NIH Toolbox DCCS and Flanker tasks (Zelazo et al., 2013). The Flanker task follows a similar format as the ANT task used at Time 1 except that the cue was always a central star and stimuli were arrows instead of fish. Participants completed 4 practice trials and 20 test trials. The DCCS was the same format as at Time 1: participants again sorted stimuli by shape (dominant) or color (non-dominant). They completed eight practice trials (four sorting by shape and four sorting by color) and 30 test trials, which included 80% dominant cues and 20% non-dominant cues. A combined score that took into account accuracy and reaction times was calculated for each of the two tasks (theoretical range 0–10).

## Results

We first examined effects of age and gender on variables of interest performance on EF tasks, P300, SPN, sensation seeking, academic performance, and social skills. We then examined stability of cool EF performance from age 8–12, followed by concurrent and longitudinal links between cool EF and adaptive behavior. Finally, we examined whether performance on the HDT task and neural correlates predicted later outcomes.

### Preliminary analyses

We examined descriptive statistics for the Flanker and DCCS at age 8 and 12 (Table 1). At age 8, reaction times for incongruent/non-dominant trials were negatively correlated with accuracy, indicating that children slowed down to perform well on the tasks at this age. Therefore, we used percent accuracy on these more difficult trials as predictors of future EF and adaptive behavior. For age 12, accuracy scores reached ceiling, so we used a composite of accuracy and RT using the NIH toolbox algorithm.

**Table 1:** **Descriptive statistics for executive function variables**.

	***N***	**Theoretical range**	**Mean**	**Std. Dev**
Age 8: Flanker incongruent accuracy	75	0–1	0.95	0.07
Age 8: DCCS non-dominant accuracy	77	0–1	0.83	0.11
Age 12: Flanker score	67	0–10	7.79	0.45
Age 12: DCCS score	67	0–10	7.28	0.63

*Note: Only children who participated at both time points are included*.

We examined whether verbal ability (assessed at age 8 using the *PPVT-4*) was correlated with EF performance and questionnaire scores. Verbal ability was not significantly correlated with any variables of interest (*r*'s = −0.002 to 0.25). However, it was marginally correlated with academic performance (*r* = 0.25, *p* < 0.09), so we controlled for verbal ability when examining correlations with academic performance.

In addition, we examined gender differences for each variable. Girls obtained significantly higher scores on the DCCS [*F*_(1, 65)_ = 3.06, *p* < 0.01] and self-reported better social skills [*F*_(1, 75)_ = 6.5, *p* < 0.02] at age 12. No other significant gender differences were found.

### Stability of EF from age 8 to 12

To examine the stability of EF, we included only the 67 children who completed at least one EF task at both time points. At age 8, Flanker and DCCS performance were measured using accuracy on incongruent and non-dominant (color) trials, respectively. Accuracies for incongruent/non-dominant trials on the two tasks were not significantly correlated at this age [*r*_(66)_ = 0.20, *p* = 0.1], although this is likely due to a ceiling effect on Flanker task accuracy at age 8. Reaction times were significantly correlated across the two tasks [*r*_(66)_ = 0.29, *p* < 0.02]. RTs were positively correlated with accuracy [Flanker: *r*_(66)_ = 0.27, *p* < 0.03; DCCS: *r*_(66)_ = 0.41, *p* = 0.001], indicating that children at this age slowed down to achieve better performance, whereas at later ages, faster RTs indicate greater efficiency.

At age 12, accuracy on these tasks reached ceiling levels, so performance was measured using an algorithm from the NIH toolbox (Zelazo et al., 2013) that combined accuracy and reaction time (in which participants receive a higher score for responding quickly after full accuracy is reached). This algorithm computes a score from 0 to 10 (sample range = 5–8.67). At age 12, performance on the DCCS and Flanker were uncorrelated, *r*_(67)_ = 0.015. As shown in Table 2, age 8 Flanker accuracy predicts age 12 Flanker, but not DCCS performance, while age 8 DCCS accuracy predicts age 12 DCCS, but not Flanker performance. RTs at age 8 were not significantly correlated with performance at age 12 (*p*s > 0.08). These results indicate longitudinal stability within but not across each EF task.

**Table 2:** **Correlations between age 8 and age 12 variables**.

	**Age 8 Flanker (ANT)**	**Age 8 HDT P3 Effect**	**Age 8 HDT SPN Effect**	**Age 8 HDT Losses**	**Age 12 DCCS**	**Age 12 Flanker**	**Age 12 TAS**	**Age 12 Child Soc. Skills**	**Age 12 Teacher Soc. Skills**	**Age 12 Academic Perf. (PPVT Control)**
Age 8 DCCS	0.216	0.108	−0.030	−0.089	0.278^*^	−0.123	−0.210	0.016	−0.133	−0.218(0.069)
	*n* = 75	*n* = 74	*n* = 75	*n* = 76	*n* = 66	*n* = 66	*n* = 75	*n* = 75	*n* = 66	*n* = 62(52)
Age 8 Flanker (ANT)	–	0.005	−0.026	−0.21	−0.059	0.381^**^	−0.085	0.027	0.139	0.35^**^(0.35)^**^
		*n* = 72	*n* = 73	*n* = 74	*n* = 64	*n* = 64	*n* = 75	*n* = 75	*n* = 64	*n* = 62(52)
Age 8 HDT P3 Effect		–	0.144	−0.111	0.166	−0.037	−0.250^*^	−0.123	0.102	0.061(0.053)
			*n* = 74	*n* = 74	*n* = 63	*n* = 63	*n* = 73	*n* = 73	*n* = 65	*n* = 62(44)
Age 8 HDT SPN Effect			–	−0.074	−0.080	−0.007	0.091	−0.148	0.139	0.281^*^(0.31)^*^
				*n* = 75	*n* = 63	*n* = 63	*n* = 73	*n* = 73	*n* = 65	*n* = 62(45)
Age 8 HDT Losses				–	−0.072	0.077	0.104	−0.001	0.065	−0.17(−0.18)
					*n* = 65	*n* = 65	*n* = 75	*n* = 75	*n* = 66	*n* = 62(44)
Age 12 DCCS					–	0.015	−0.071	0.292^*^	−0.052	−0.32^*^(−0.33)^*^
						*n* = 67	*n* = 63	*n* = 64	*n* = 54	*n* = 51(48)
Age 12 Flanker						–	0.072	−0.086	0.032	−0.106(−0.142)
							*n* = 63	*n* = 63	*n* = 54	*n* = 51(48)
Age 12 TAS							–	−0.174	−0.03	0.151(0.144)
								*n* = 77	*n* = 62	*n* = 58(55)
Age 12 Child Soc. Skills								–	0.348^**^	0.191(0.161)
									*n* = 62	*n* = 58(55)
Age 12 Teacher Soc. Skills									–	0.61^**^(0.58)^**^
										*n* = 62(55)

* p < 0.05,

** *p < 0.01*.

*Correlations are computed based on children who participated at both age 8 and age 12. Correlations involving academic competence are presented both raw and partial (controlling for PPVT score). TAS, Thrill/Adventure Seeking*.

### Concurrent links between EF and adaptive behavior at age 12

We examined links between our EF measures and self- and teacher-reported social skills, thrill/adventure seeking (self-report only), and academic competence (teacher report only) at age 12. DCCS performance was positively correlated with self-reported social skills, *r*_(64)_ = 0.29, *p* = 0.02, but negatively correlated with academic competence *r*_(42)_ = −0.32, *p* = 0.04 (Table 2). Because there was an effect of gender on DCCS performance at age 12, we performed a partial correlation controlling for gender, which did not affect the magnitude of these correlations. Flanker performance was not significantly correlated with any outcome variables.

### Links between age 8 EF and age 12 adaptive behavior

We tested the degree to which accuracy on EF tasks at age 8 predicts adaptive behavior at age 12. Flanker incongruent trial accuracy significantly predicted teacher-rated academic competence, but no other outcome variables. This correlation remained significant when controlling for verbal ability and age 12 Flanker performance (see Table 2). DCCS non-dominant trial accuracy did not predict any outcome variables.

### Longitudinal predictions of affective decision-making

Next, data were analyzed to assess the degree to which performance on and neural correlates of a risky decision-making task at age 8 (Hungry Donkey) predicted individual differences in variables of interest at age 12. To measure performance, we examined the total number of trials in which a net loss was incurred. Two neural correlates were of interest, stemming from our previous findings (Carlson et al., 2009): (i) the magnitude of the post-stimulus P300 and (ii) the pre-stimulus/anticipatory SPN components in response to reward and loss trials.

The P300 was of significantly larger magnitude in response to loss vs. reward trials, [*F*_(1, 78)_ = 31.2, *p* < 0.001] and the SPN was significantly larger (more negative-going) after high-frequency loss door selections than low-frequency loss door selections [*F*_(1, 78)_ = 6.51, *p* < 0.02]. However, our primary question was whether individual differences in the magnitude of P300 to loss trials and SPN to high-frequency loss door selections predicted adaptive outcomes at age 12. Individual differences in P300 effect (magnitude to loss-minus-reward trials) was negatively correlated with self-reported thrill/adventure seeking. In other words, the larger the neural response to punishment (vs. reward) outcomes at age 8, the less likely participants were to report an interest in thrill/adventure 4 years later in pre-adolescence. The P300 effect was not significantly correlated with other outcome variables.

In addition, individual differences in SPN magnitude to low-minus-high-frequency loss doors, in which more positive values reflect pre-outcome anticipation of loss, significantly predicted academic competence at age 12, even when controlling for verbal ability. This finding suggests that a neural correlate of risk-aversion at age 8 was related to higher academic competence at age 12, but not to other outcome variables. The P300 and SPN components did not predict EF performance at age 12, and the loss count on the HDT task did not predict any outcomes at age 12 (see Table 2 for summary).

## Discussion

The goals of this research were to examine the stability of EF and the extent to which individual differences in neural responses to affective cues serve as a biological marker for EF and adaptive behaviors in a sample followed longitudinally from age 8 to 12. Two broad findings emerged. We found that (i) cool aspects of EF showed modest stability from middle childhood to early adolescence and that (ii) certain aspects of childhood cool EF and neural sensitivity to reward and punishment predicted some individual differences in sensation seeking and adaptive behaviors in children entering adolescence.

### Stability of cool EF and links to adaptive behavior

To our knowledge, this is the first study to demonstrate longitudinal stability of performance on specific EF tasks in this age range from middle childhood to adolescence. Our findings add to a growing body of evidence suggesting that individual differences in EF remain stable beyond the preschool period (Eigsti et al., 2006; Casey et al., 2011). In our sample, the inhibition (ANT/Flanker) and shifting/updating (DCCS) aspects of EF showed stability across the age range tested: Individual differences in ANT performance at age 8 predicted Flanker performance at age 12, and DCCS performance at age 8 predicted DCCS performance at age 12. However, performance on these two tasks were uncorrelated with each other in this age range. Given that different dimensions of cool EF tend to be strongly correlated in the preschool years (e.g., Wiebe et al., 2011), our findings suggest that dimensions of cool EF may become more differentiated over time, which is compatible with prior cross-sectional work reporting separability of working memory updating and inhibitory control beginning around age 9–10 (Shing et al., 2010). Taken together, these results support an idea of increasing specialization of circuits within the PFC for specific cognitive functions across development (e.g., Zelazo and Carlson, 2012).

A noteworthy aspect of our longitudinal design is that we documented that inhibitory control (ANT) at age 8 predicted teacher-rated academic competence at age 12. This finding fits with other work linking EF and later academic achievement in a variety of age groups (Blair and Razza, 2007; Best et al., 2011). Links between early EF and later academic performance make sense, given that the abilities to inhibit prepotent responses and to ignore distractions are necessary to develop the self-control necessary to be attentive in class and to study or do homework instead of engaging in other activities. The fact that this finding was independent of verbal ability (PPVT) lends further support to the emerging belief that EF matters for later academic achievement over and above, and perhaps more than IQ (for review, see Duckworth and Carlson, 2013).

### Neural sensitivity to punishment vs. reward predicts later behavior

Neural responses during the child-friendly gambling task (a hot EF task) at age 8 predicted a variety of adaptive behaviors at age 12. Greater sensitivity to loss trials and high-frequency loss doors, as indexed by the magnitude of P300 and SPN difference scores, predicted lower propensity for thrill/adventure seeking and better academic outcomes, respectively. Interestingly, avoidance of losses during the task did not correlate with later outcomes, suggesting that our ERP measures were more sensitive than behavior. Although children showed sensitivity to rewards and punishments at a neural level, they may not have been fully able to translate that sensitivity into improved performance during the course of the task. However, our results suggest that greater sensitivity to punishment vs. reward may play an important role in the development of children's trajectories toward more cautious/conscientious vs. higher risk-taking behavioral patterns.

Children who had shown attenuated P300 amplitudes after loss trials relative to reward trials reported more desire to engage in risky behaviors in early adolescence. This finding fits well with results linking reduced P300 amplitude to risky behaviors such as alcohol use in adolescence (Carlson et al., 1999; McGue et al., 2001). Although ours was a low-risk sample that reported low levels of externalizing behaviors in general, these findings add to evidence that the P300 could be a psychophysiological marker linked to propensity for risk-taking and/or sensation seeking. These findings also suggest that children who devoted more attentional resources to loss trials (reflected in *higher* P300 amplitudes) may tend toward more risk-averse behavioral trajectories.

While the post-feedback P300 at 8 years old was associated with adolescent thrill/adventure seeking, it is interesting that pre-feedback SPN at 8 years old predicted academic outcomes in adolescence. Specifically, we found that greater magnitude of SPN responses to high-frequency loss doors predicted greater academic success. The SPN is believed to index a “somatic marker” (Damasio, 1996) involving cortical and subcortical activity related to the expectation for relevant positive or negative feedback (Brunia et al., 2011). For example, it is enhanced under conditions in which outcomes are linked to actions vs. occurring at random (Masaki et al., 2010), suggesting that a sense of control during a task is necessary to elicit the SPN. In our study, SPN responses tended to be larger just before high-probability loss outcomes than low-probability loss outcomes. On the surface, our findings might seem to contradict those of Stavropoulos and Carver (2013), who found that anticipation of more socially rewarding vs. less rewarding feedback elicited a larger SPN in children. However, both findings make sense if SPN is a marker of the salience or motivational relevance of stimuli. While the former study did not involve punishment, ours did, so children were likely motivated primarily to avoid losses. In the current study, children who showed a larger SPN in the moment just after making a risky selection and just before a loss outcome was revealed might be more sensitive to the fact that they made a non-optimal response and therefore expect to receive negative feedback. In other words, they had a “feeling” or intuition detectable at a neural level that they were about to suffer a loss on the next trial. The present study suggests that perhaps they felt they had agency to avoid future losses and were better able to learn from mistakes in general, which may in turn facilitate learning from pedagogical instruction and success in school.

This interpretation about the SPN is speculative, in part because the interpretation and study of SPN is just beginning to be applied in children (e.g., Stavropoulos and Carver, 2013). However, a cross-sectional study recently showed that activity in insular cortex, believed to be a primary generator of the SPN, increased between age 5 and adulthood during a gambling task, corresponding to an age-related increase in risk aversion (Paulsen et al., 2011). In addition, emerging evidence links the SPN to the dopaminergic learning systems believed to underlie the error-related negativity component (ERN) in adults (Moris et al., 2013). The ERN varies in magnitude according to the difference between expected and received feedback and has been better characterized developmentally than the SPN. Examining both of these components in development and linking them to laboratory and real-world behavior would yield rich information about how neural sensitivity to reward and punishment relates to learning and adaptation.

### Limitations and future directions

This research has limitations and suggests future directions. Although we found that cool EF and ERP components during a hot EF task predicted some individual differences 4 years later, we found no evidence to support other predicted longitudinal relations. Behavioral performance on the HDT did not predict later outcomes, suggesting that on this task, ERP components were a more sensitive measure of individual differences in sensitivity to reward and punishment. However, these neural measures did not predict later social skills, which were related (for self-report) only to concurrent DCCS performance. Surprisingly, at the same time, age 12 DCCS performance was *negatively* correlated with teacher-rated academic performance. Self-rated social skills and teacher-rated academic competence were not significantly correlated, which is not surprising at this age in a low-risk sample. Possibly, children in our sample who are more successful academically were more conscientious in general, and this negatively affected their score on the DCCS, where slowing down in order to be accurate would result in a cost. We also found no evidence that cool EF at either time point predicted thrill/adventure seeking, but this null finding fits with developmental literature showing evidence of a dissociation between impulsivity and sensation seeking (Steinberg et al., 2008).

Another limitations is that our sample was relatively homogeneous in terms of ethnic and socioeconomic background. Children were generally low-risk and not (yet) endorsing many of the substance use and social risk-taking behaviors on the SSSC. We are continuing to follow this cohort through adolescence when some of these items will become more sensitive. Nonetheless, there were sufficient individual differences in academic competence and thrill/adventure seeking (e.g., enjoyment of riding one's bike fast down a steep hill) to detect longitudinal predictions from 4 years earlier. As well, we did not use the same versions of the EF tasks at age 8 and 12 in this longitudinal sample because the abbreviated NIH Toolbox versions were not available at Time 1 and developed in the interim. Nevertheless, the within-task stability of the Flanker and DCCS was significant. Finally, given our relatively small sample size, correction for multiple comparisons would have reduced some findings to non-significance, but we note that we were selective in our comparisons and had a priori hypotheses regarding each of them.

Despite these limitations, this is the first longitudinal study, to our knowledge, to examine the development of both hot and cool EF and their relations to adaptive behavior between middle childhood and pre-adolescence. We found that individual differences in performance on EF tasks were stable across this age range and that certain aspects of cool and hot EF predicted individual differences in thrill/adventure seeking and academic outcomes at age 12.

These novel findings generate many potential directions for future research, especially regarding adolescent brain development and the prediction of individual differences in adaptive behavior. Hot and cool EF appear to interact in complex ways that change across development and these aspects of EF may relate to behavior differently in childhood, adolescence, and adulthood. For example, hot EF may become increasingly important relative to cool EF in adolescence, when individuals begin to take greater control of their environment and make more decisions for themselves. In addition, adolescents show more sensitivity to social facilitation from peers than children or adults in the context of a risky decision-making task (Gardner and Steinberg, 2005). Therefore, we might expect peers to play a larger role in either facilitating or hindering adaptive behavior in adolescence than in other age groups. With this in mind, both social understanding and EF may be key to the successful navigation through adolescence. Future research could also more deeply explore the relations between EF and risk aversion, as opposed to risk-taking. Such work could have implications for anxiety disorders, which may be linked to maladaptive levels of risk aversion (Robin and Martin, 2010) and are especially prevalent in the teenage years. Exploration of the role of hot EF in development, particularly at a neural level, is a new area that holds great promise for deepening our understanding of human brain-behavior relations, and we expect that future studies will yield information with high applicability for developmental theory, educational practice, and clinical science.

## Author contributions

Madeline B. Harms wrote the majority of the manuscript and contributed to the study design and data collection at Time 2. Vivian Zayas contributed to the study design and data collection at Time 1. This research was conducted in the lab of Stephanie M. Carlson and she contributed to the study design and data collection at both time points. All four authors contributed to analysis and interpretation of data and revising the manuscript, gave final approval of the version to be published, and agree to be accountable for all aspects of the work.

### Conflict of interest statement

The authors declare that the research was conducted in the absence of any commercial or financial relationships that could be construed as a potential conflict of interest.
